# Maternal HIV infection drives altered placental *Mtb*-specific antibody transfer

**DOI:** 10.3389/fmicb.2023.1171990

**Published:** 2023-05-09

**Authors:** Nadege Nziza, Wonyeong Jung, Maanasa Mendu, Tina Chen, Ryan P. McNamara, Sarah M. Fortune, Kees L. M. C. Franken, Tom H. M. Ottenhoff, Bryan Bryson, Joseph Ngonzi, Lisa M. Bebell, Galit Alter

**Affiliations:** ^1^Ragon Institute of MGH, MIT and Harvard, Cambridge, MA, United States; ^2^Department of Molecular and Cellular Biology, Harvard University, Boston, MA, United States; ^3^Department of Immunology and Infectious Diseases, Harvard T.H. Chan School of Public Health, Boston, MA, United States; ^4^Department of Infectious Diseases, Leiden University Medical Center, Leiden, Netherlands; ^5^Department of Biological Engineering, Massachusetts Institute of Technology, Cambridge, MA, United States; ^6^Department of Obstetrics and Gynecology, Mbarara University of Science and Technology, Mbarara, Uganda; ^7^Division of Infectious Diseases, Massachusetts General Hospital, Boston, MA, United States; ^8^Medical Practice Evaluation Center, Massachusetts General Hospital, Boston, MA, United States; ^9^Center for Global Health, Massachusetts General Hospital, Boston, MA, United States

**Keywords:** antibody, placental transfer, neonate, HIV, tuberculosis

## Abstract

**Introduction:**

Placental transfer of maternal antibodies is essential for neonatal immunity over the first months of life. In the setting of maternal HIV infection, HIV-exposed uninfected (HEU) infants are at higher risk of developing severe infections, including active tuberculosis (TB). Given our emerging appreciation for the potential role of antibodies in the control of *Mycobacterium tuberculosis* (*Mtb*), the bacteria that causes TB, here we aimed to determine whether maternal HIV status altered the quality of *Mtb*-specific placental antibody transfer.

**Methods:**

Antigen-specific antibody systems serology was performed to comprehensively characterize the *Mtb*-specific humoral immune response in maternal and umbilical cord blood from HIV infected and uninfected pregnant people in Uganda.

**Results:**

Significant differences were noted in overall antibody profiles in HIV positive and negative maternal plasma, resulting in heterogeneous transfer of *Mtb*-specific antibodies. Altered antibody transfer in HIV infected dyads was associated with impaired binding to IgG Fc-receptors, which was directly linked to HIV viral loads and CD4 counts.

**Conclusions:**

These results highlight the importance of maternal HIV status on antibody transfer, providing clues related to alterations in transferred maternal immunity that may render HEU infants more vulnerable to TB than their HIV-unexposed peers.

## Introduction

During the first months of life, protection against infectious diseases relies on the transfer of maternal immunity across the placenta (Levy et al., 2013; Dowling and Levy, 2014; Jennewein et al., 2019). In healthy pregnancy, IgG levels in the umbilical cord blood typically are enriched, reaching more than 100% of maternal IgG levels (Kohler and Farr, 1966; Palmeira et al., 2012), due to the active transfer of antibodies across the placenta. However, in the setting of chronic maternal diseases, placental antibody transfer can be compromised, resulting in altered or incomplete transfer of immunity to the neonate, rendering infants vulnerable early in life (Palmeira et al., 2012). Along these lines, each year, more than 1 million HIV-exposed uninfected (HEU) children are born, with a 4-fold higher risk of severe infection, morbidity and mortality compared to HIV-unexposed infants (Slogrove et al., 2012; Cotton et al., 2014; Labuda et al., 2020). Studies have shown conflicting results concerning the impact of maternal HIV status on the transfer of antibodies, with both evidence of impaired and unchanged transplacental transfer of antibodies in pregnant people with HIV (Jones et al., 2011; Jallow et al., 2017; Ho et al., 2019; Dolatshahi et al., 2022b).

Tuberculosis (TB) is among the infections associated with the greatest risk of mortality in early life, as infants struggle to contain TB, with high rates of both active pulmonary and disseminated TB (Newton et al., 2008; Vanden Driessche et al., 2013; Thomas, 2017). In fact, while approximately 5% of adults infected with TB have a lifetime risk of developing active TB disease, an estimated 50% of infants develop active disease immediately after infection (Basu Roy et al., 2019). Moreover, HEU infants are at even higher risk of developing TB, with an estimated 4-fold increased risk of active disease compared to infants not exposed to HIV (Madhi et al., 2011; Ruck et al., 2016). Whether infant vulnerability relates to the naïve state of the child’s immune system, HIV-associated inflammatory imprinting across the dyad, or due to alterations in other immune factors such as maternal antibody transfer remains poorly understood.

Using an unbiased and antigen-specific systems serology approach, we comprehensively profiled the *Mtb*-specific humoral response in maternal and umbilical cord blood across 209 *Mtb* antigens in HIV-infected as well HIV-uninfected pregnant people living in Uganda and their placental umbilical cords. We present data highlighting the significant impact of maternal HIV infection on placental transfer of *Mtb*-specific antibodies, with a shift toward reduced inflammatory antibody transfer ratios. We also observed a strong positive correlation between antibody transfer profiles and maternal CD4 T cell (CD4) count, and negative correlation between antibody transfer and maternal viral load. This suggests that more robust maternal immune reconstitution may compensate for HIV-associated inflammation and altered antibody transfer, in a viral load and CD4 count dependent manner, ultimately impacting infant immunity.

## Materials and methods

### Study subjects

Pregnant people with HIV (*n* = 10) or without HIV (*n* = 14) were recruited from Mbarara Regional Referral Hospital in Uganda in 2017–2018 and maternal plasma was collected in labor and umbilical cord blood was collected within 30 min of placental delivery (Table 1). HIV-infected people all reported taking antiretroviral therapy (ART). TB is highly endemic in East Africa, such that all participants were considered highly exposed to *Mtb*, with evidence or confirmed diagnosis for active forms of the disease when the participants presented to hospital in labor for delivery. HIV viral load and/or CD4 count was measured for HIV-infected people who did not have a record of these measurements within the last 6 months. The project was approved by Mbarara University of Science and Technology (11/03–17) and Partners Healthcare (2017P001319).

**Table 1:** Demographic, medical, and placental characteristics of a cohort of people presenting in labor to Mbarara Regional Referral Hospital in Uganda, by HIV serostatus.

Characteristic	HIV-infected (*n* = 10)	HIV-uninfected (*n* = 14)	*p*-value*
Demographics			
Age category			0.11
≤19	0 (0)	1 (7)	
20–34	10 (100)	9 (64)	
≥34	0 (0)	4 (29)	
Resides in Mbarara district	6 (60)	11 (79)	0.32
Married	10 (100)	13 (93)	0.39
Monthly income^a^ (median USD^b^, IQR^c^)	$20 (12, 49)	$39 (21, 91)	0.08
Formal employment outside the home	2 (20)	11 (79)	0.005
Obstetric and medical			
Gestational age in weeks (mean, SD^d^)	39 (1.8)	40 (1.9)	0.45
Parity prior to current delivery			0.21
0 (primiparous)	1 (10)	6 (43)	
1–3 (multiparous)	5 (50)	4 (29)	
>4 (grand multiparous)	4 (40)	4 (29)	
Attended ≥4 antenatal care visits this pregnancy	4 (40)	8 (57)	0.41
Cesarean delivery	2 (20)	2 (14)	0.71
Days hospitalized for delivery (mean, SD)	2.2 (2)	2.2 (3)	0.73
Birthweight category (in kilograms)			0.10
<2.5	1 (10)	0 (0)	
2.5–3.5	9 (90)	8 (62)	
3.6–4.0	0 (0)	4 (31)	
>4.0	0 (0)	1 (8)	

*Tests of association between cohort characteristics and HIV serostatus were performed using Fisher’s exact, Wilcoxon rank sum, and *t*-tests. ^a^Reported by participant in Ugandan Shillings, converted to ^b^USD (United States Dollar) using exchange rate for study start date (March 1, 2017: 1 USD = 3,600 Ugandan Shillings); ^c^IQR: interquartile range; ^d^SD: standard deviation. Results listed as “*n* (%)” unless otherwise noted.

### Antigens

Two hundred and seven *Mtb* antigens, selected based on their discriminatory potential for TB diagnosis and their immunogenicity (Franken et al., 2000; Serra-Vidal et al., 2014; Coppola and Ottenhoff, 2018) were obtained from Dr. Tom Ottenhoff and Kees Franken (Nziza et al., 2022), and prepared as previously described (Franken et al., 2000; Commandeur et al., 2013). Purified LAM was obtained from BEI Resources and purified protein derivative (PPD) was received from the Statens Serum Institute. Influenza Virus A, pertussis and gp120 were purchased from ImmuneTech.

### Antibody isotype, subclass and FcR binding

Antigen-specific antibody isotype and subclass levels, as well as Fcy-receptor (FcyR) binding were quantified in maternal and umbilical cord blood plasma samples by Luminex multiplexing, as previously reported (Brown et al., 2012). Antigens were coupled to distinct magnetic Luminex beads through carbodiimide-NHS ester coupling. Maternal and umbilical cord blood plasma (dilution 1:100) samples were then added to the bead/antigen mixes to form immune complexes. Immune complexes were washed after a 2-h incubation at room temperature, and antibody levels were detected using phycoerythrin (PE)-conjugated mouse anti-human IgG1, IgG3 or IgM (Southern Biotech) at 1.3 ug/mL. FcyRs were purchased from Duke Human Vaccine Institute, and *Mtb*-specific antibody binding to FcyR2A, FcyR2B, FcyR3A and FcyR3B was measured using PE-streptavidin (Agilent Technologies). After incubation with subclasses/isotypes or FcyRs, median fluorescence intensity (MFI) from immune complexes was determined on an iQue analyzer (Intellicyt).

### Antibody-dependent neutrophil phagocytosis

Antibody-dependent neutrophil phagocytosis (ADNP) was assessed with a microsphere-based phagocytic assay, as described previously (Karsten et al., 2019). Immune complexes, composed of antigen-coupled neutravidin microspheres (Thermo Fisher F8776) and antibodies from plasma and umbilical cord blood samples (dilution 1:100), were formed, then human neutrophils from healthy donors’ blood were added and incubated at 37°C. Neutrophils were stained with anti-Cd66b Pac blue antibody (BioLegend 305,112) and fixed with 4% paraformaldehyde (PFA). Analysis was performed by flow cytometry, and phagocytosis score was calculated as the (percentage of microsphere-positive Cd66b cells) × (MFI of microsphere-positive cells) divided by 100,000.

### Antibody-dependent cellular phagocytosis

For antibody-dependent cellular phagocytosis (ADCP), THP-1 phagocytosis was measured as previously described (Ackerman et al., 2011). Antigens were coupled to yellow-green fluorescent neutravidin microspheres (Thermo Fisher F8776), then incubated with maternal or umbilical cord plasma samples (dilution 1:100). After a 2-h incubation, THP-1 monocytes (0.25 M cells per well) were added and incubated for 16 h at 37°C. After fixing the cells with 4% paraformaldehyde, analyses were conducted by flow cytometry, and phagoscore was calculated as the (percentage of microsphere-positive cells) × (MFI of microsphere-positive cells) divided by 100,000.

### Statistical and computational analyses

GraphPad software version 8.0 was used for the visualization and the analyses of all univariate plots. Plots show the average of two replicates for both Luminex and functional assays. Paired samples Wilcoxon test was used to identify significant differences between maternal and umbilical cord blood plasma. For the analyses of transfer ratios, umbilical cord blood plasma antibodies were divided by maternal plasma antibodies. Volcano plots that illustrate the correlation between antibody response and maternal HIV viral load and CD4 count were built using Spearman correlations (*p* < 0.05), with 84 HIV-infected women.

In order to identify features that discriminated HIV-positive (with HIV-infected/exposed dyads, *n* = 10) and HIV-negative (with HIV-uninfected/unexposed dyads, *n* = 14) pregnancies, a multivariate approach was used, which combined a least absolute shrinkage and selection operator (LASSO) for feature selection, as well as a partial least square discriminant analysis (PLS-DA) using the LASSO selected features (Lau et al., 2011; Belkina et al., 2018). Model accuracy was measured using five-fold cross-validation. The selection of LASSO-selected features was repeated 100 times for each test, and the features that were selected a minimum of 90 times out of 100 were designated as selected features. Co-correlates analyses were performed with LASSO selected features to visualize additional antibody features that were involved in the discrimination between HIV positive and negative pregnancies. Network plots were used to represent the co-correlates that were identified using Spearman’s method followed by Benjamini-Hochberg correction, including only features with coefficients >0.75 and *p* value <0.05.

## Results

### *Mtb*-specific antibody profiles in maternal and umbilical cord blood plasma are impacted by maternal HIV status

**Figure 1:**
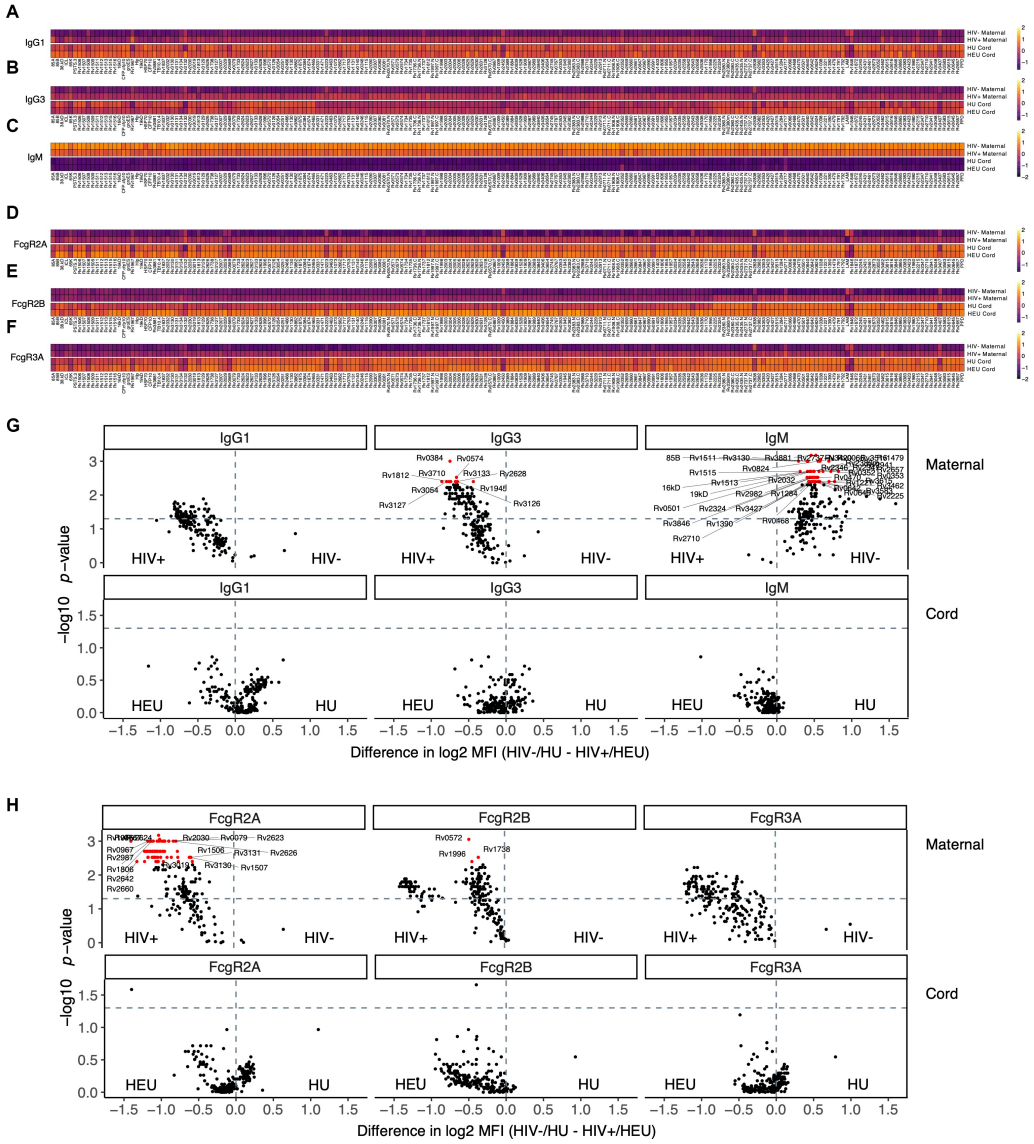
Maternal HIV infection has a stronger impact on plasma *Mtb*-specific antibody response compared to umbilical cord blood. Antibody levels against 209 *Mtb* antigens and their capacity to bind to FcR were measured in the plasma of HIV infected and uninfected people, in addition to their exposed/unexposed umbilical cord blood. **(A–F)** Heatmaps show median of Z-scored MFI values for IgG1 **(A)**, IgG3 **(B)** and IgM **(C)**, as well as FcyR2A **(D)**, FcyR2B **(E)** and FcyR3A **(F)** binding. **(G,H)** Volcano plots illustrate the difference between HIV negative and HIV positive pregnancies for isotypes **(G)** and FcR binding **(H)**. The magnitude of the difference is on the x-axis, and the significance (*p* values) on the y-axis. Values above the horizontal dashed lines are significantly different between HIV infected/exposed and HIV uninfected/unexposed conditions (*p* < 0.05). Red features have an adjusted *p* value <0.05 by the Benjamini–Hochberg procedure.

To begin to determine whether HIV status influences maternal and umbilical cord *Mtb*-specific humoral profiles, we initially aimed to capture antibody profiles against 8 common *Mtb* antigens (LAM, PPD, Ag85A and 85B, PSTS3, ESAT6/CFP10 and groES) as well as 201 additional *Mtb* antigens that were transcriptionally highly active in *Mtb* infected lungs (Commandeur et al., 2013; Nziza et al., 2022). *Mtb* specific antibody isotypes, subclasses, Fc-receptor binding, and antibody functionality were analyzed in maternal and umbilical cord blood plasma for both HIV-infected maternal/HEU and HIV-uninfected maternal/HIV-unexposed (HU) dyads (Figure 1). Of note, fewer participants with HIV than without HIV were primiparous (Table 1). This is a common finding in studies comparing people with and without HIV infection and is likely related to the common route of infection with HIV through sexual activity. However, there are no expected impacts on our findings based on the difference in parity. Antigen-specific transplacental antibody transfer does not vary significantly with change in parity. Additionally, IgG1 responses were higher in the HIV-infected group compared to HIV-uninfected pregnant people for most tested *Mtb* antigens (Figures 1A,G), likely related to the hypergammaglobulinemia associated with HIV infection (De Milito et al., 2004; van Woudenbergh et al., 2020). Moreover, IgG1 levels were higher in umbilical cord plasma for both HEU and HU samples compared to their respective maternal plasma profiles (Figure 1A), consistent with known enrichment of antibodies in neonatal blood during placental transfer. Surprisingly, more IgG1 was not observed in the cord samples from HEU compared to HU (Figures 1A,G), suggesting that the placenta may tightly regulate the amount of antibody transferred even in the setting of maternal hypergammaglobulinemia. Moreover, for all antigens, HEU and HU umbilical cord antibody profiles were relatively similar. *Mtb*-specific IgG3 responses showed a similar pattern (Figure 1B), with strong differences between HIV-infected and -uninfected pregnant people (Figure 1G). Also, elevated transfer of IgG3 was noted into the umbilical cord for most *Mtb*-specific antigens at equal levels for both HEU and HU umbilical cord samples (Figures 1B,G). In contrast, IgM displayed a starkly different pattern, with a significant enrichment in HIV-uninfected maternal plasma compared to plasma from HIV-infected people, and no measurable transfer to the umbilical cord (Figures 1C,G), as expected due to the lack of a transfer mechanism for IgM across the placenta (Jennewein et al., 2017). Finally, the capacity of antibodies to bind to low affinity IgG Fc-receptors (FcRs), that govern innate immune effector functions (Jennewein and Alter, 2017), were also evaluated (Figures 1D–F,H). Similar to IgG profiles, a strong binding to FcγR2A, FcγR2B and FcγR3A was observed for most *Mtb* antigens in HIV-infected women. Additionally, FcR binding profiles were similar and enriched in umbilical cord blood (Figures 1D–F,H), confirming the selective transfer of FcR binding antibodies to infants to provide protection in early life (Dolatshahi et al., 2022a).

### Maternal, but not umbilical cord plasma, exhibit distinct *Mtb*-specific antibody profiles

**Figure 2:**
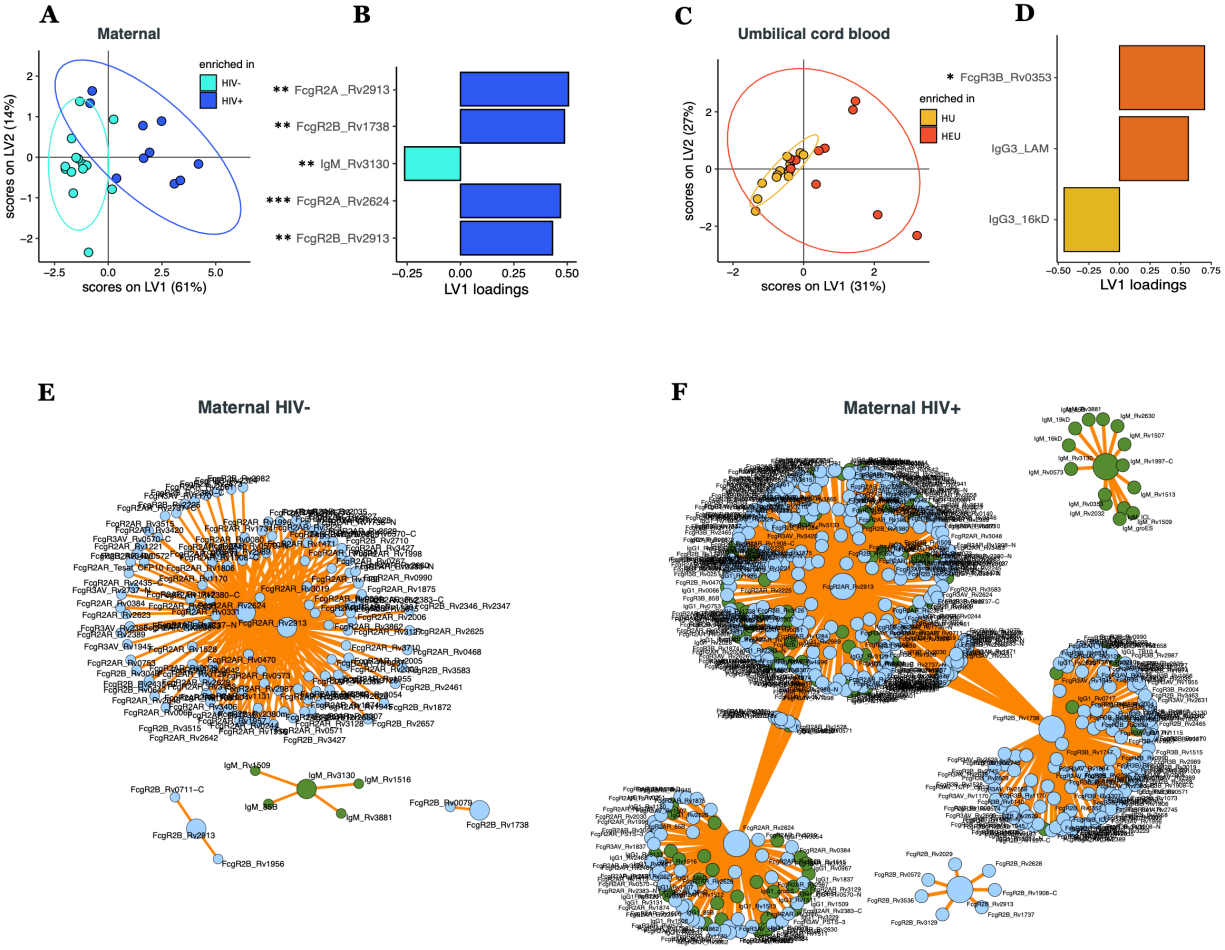
Multivariate comparison of *Mtb*-specific antibody profile in maternal and umbilical cord blood plasma in HIV-infected/exposed and HIV-uninfected/unexposed dyads. PLS-DA models showing the distribution of HIV infected and uninfected maternal plasma **(A)** and HIV exposed and unexposed umbilical cord blood **(C)**. LASSO-selected features that were used to train the PLS-DA are plotted on VIP plots **(B,D)**. LASSO-selected features significantly different between groups during univariate analyses are indicated. Significance was calculated by using Wilcoxon signed-rank test, *p < 0.05, **p < 0.01. Correlates of the LASSO-selected features are represented in network format for HIV uninfected **(E)** and HIV infected **(F)** people. Correlation networks only includes features with significant (*p* < 0.05) Spearman correlations. LASSO-selected features are on big circles, their correlates are on small circles. Green circles correspond to isotypes, blue circles correspond to FcR.

To further dissect potential multivariate effects of HIV on the cord or maternal *Mtb*-specific antibody response, we combined all the isotype, subclass, and FcR-binding data across all *Mtb*-antigens to determine whether differences existed across the antibody-profiles within maternal versus umbilical cord plasma (Figure 2). A least absolute shrinkage and selection operator (LASSO) followed by a Partial Least Squares Discriminant Analysis (PLSDA) was initially used to define a minimal combination (to avoid overfitting) of antibody-data points that provided maximal discrimination across plasma samples from HIV infected or uninfected people (Figures 2A,B, accuracy = 0.67). As few as 5 out of 1,449 total antibody features were sufficient to separate HIV-infected and uninfected maternal plasma samples (Figure 2B), marked by higher levels of FcγR2A and 2B to several *Mtb*-antigens in HIV infected people and higher RV3130-specific IgM in HIV uninfected people (Supplemental Figure S1). Furthermore, due to the conservative LASSO algorithm, that aims to select the minimal number of features that capture the greatest variance across groups, LASSO features may be co-correlated with additional antibody features that differ across the population. Thus, we next generated a co-correlates network with all 5 LASSO-selected antibody characteristics to identify the additional *Mtb*-specific humoral responses that differed across the groups. Our results revealed that the LASSO-selected antibody features were strongly linked to additional *Mtb*-specific antibody responses across both maternal populations, albeit exhibiting different overall humoral architectural profiles (Figures 2E,F). Specifically, HIV-uninfected pregnant people exhibited a single, highly coordinated cluster of antibody features. In contrast, HIV-infected pregnant people exhibited a more structured co-correlates network with distinct antibody clusters associated with each LASSO correlate. These data suggest that *Mtb*-specific antibody profiles are highly coordinated in each group, but exhibit nuanced overall humoral architectures.

In contrast to the plasma profiles, limited separation was observed in the umbilical cord antibody profiles between HEU and HU samples (Figure 2C, accuracy = 0.54). Despite this limited separation, the model selected 3 of 1,242 features to separate antibody profiles (Figure 2D), including Rv0353-specific FcyR3B binding levels and LAM-specific IgG3 that were selectively enriched in umbilical cord samples from HIV-infected people, whereas 16kD-specific IgG3 levels were selectively enriched in umbilical cord samples from HIV-negative pregnant people (Figure 2D). However, only Rv0353-specific FcyR3B binding levels were significantly different between the two umbilical cord groups (Supplementary Figure S1). Collectively, these data point to significant differences in maternal *Mtb*-specific antibody profiles, but more limited differences in cord *Mtb*-specific profiles, demonstrating the highly conserved and preserved placental antibody transfer to infants on the first days of life.

### *Mtb*-specific antibody transfer differences across HIV-infected/exposed and HIV-uninfected/unexposed dyads

**Figure 3:**
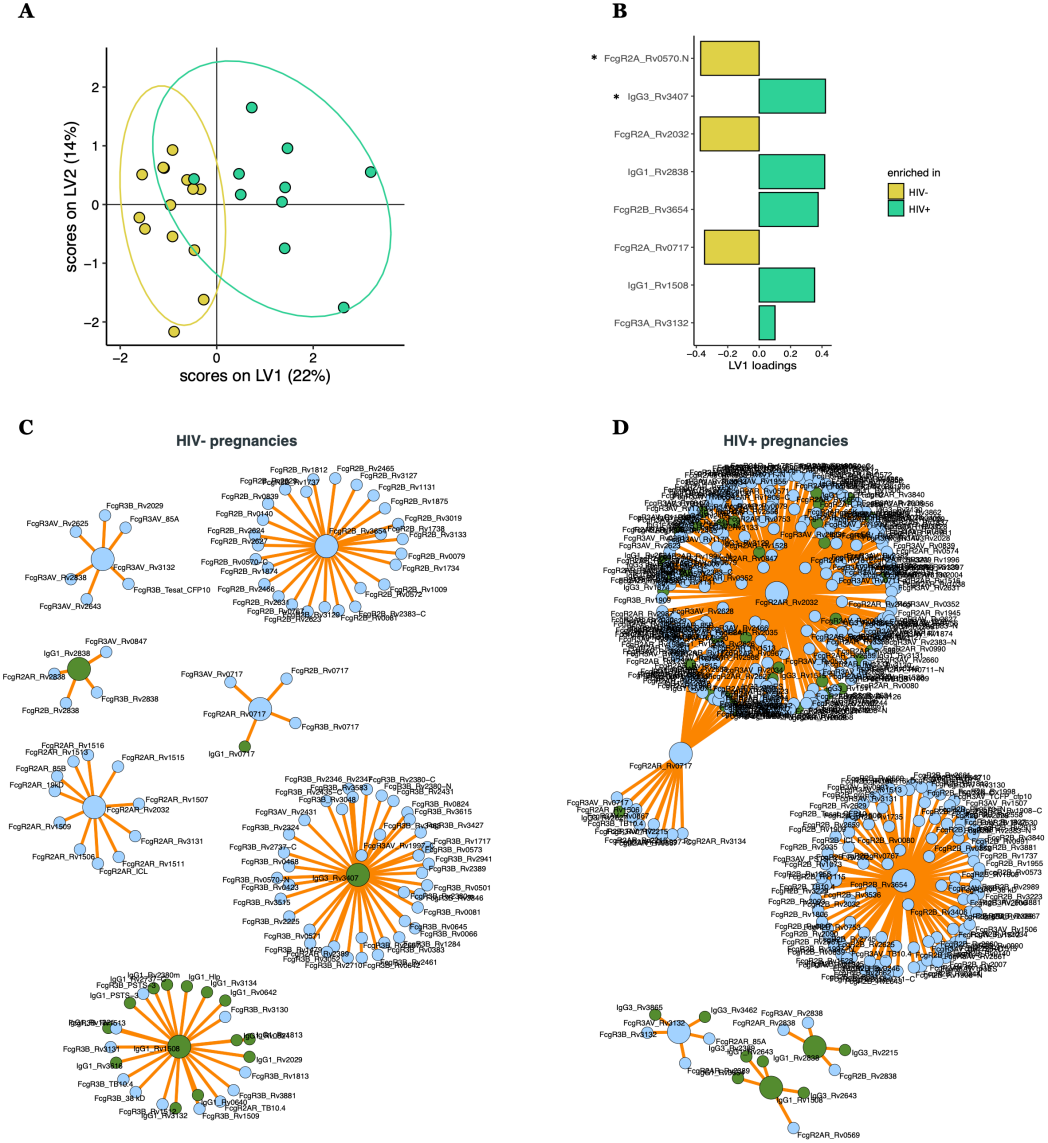
Multivariate analysis comparing transfer ratios across HIV-positive and HIV-negative pregnancies. **(A)** PLS-DA model built using LASSO-selected features **(B)**, separating HIV-positive (with HIV-infected/exposed dyads) and HIV-negative (with HIV-uninfected/unexposed dyads) pregnancies. LASSO-selected features significantly different between groups during univariate analyses are indicated. Significance was calculated by using Wilcoxon signed-rank test, *p < 0.05. **(C,D)** Correlates of the LASSO-selected features are represented in network format for HIV negative **(C)** and positive **(D)** pregnancies, including only coefficients with significant (*p* < 0.05) Spearman correlations. LASSO-selected features are on big circles, their correlates are on small circles. Green circles correspond to isotypes, blue circles correspond to FcR.

Given the striking differences in maternal, but not umbilical cord, *Mtb*-specific antibody-profiles, we next sought to define whether there were specific antibody transfer differences that could explain the mechanism(s) by which the placenta equalizes antibody transfer irrespective of the maternal profiles present at the time of birth. To achieve this goal, transfer ratios (umbilical cord/maternal plasma levels) were calculated for all antibody measurements. Comparison of transfer ratios across HIV-infected/exposed and HIV-uninfected/unexposed dyads pointed to differences in the transfer ratios across the groups (Figure 3A, accuracy = 0.65). Strikingly, HIV-uninfected/unexposed dyads exhibited a selective enrichment for FcγR2A-binding antibodies to several *Mtb*-targets (Figures 3B,C). Conversely, HIV-infected/exposed dyads appeared to selectively transfer enhanced IgG3, FcγR2B, IgG1, and FcγR3A to distinct *Mtb*-specific antibody targets (Figures 3B,D). Moreover, interactome analysis further highlighted the highly coordinated network of *Mtb*-specific antibody responses in both groups (Figures 3C,D), especially in HIV-infected/exposed dyads (Figure 3D), pointing to cross-antigen alterations in antibody transfer from pregnant people to their neonates, analogous to what has been described with other pathogen-specific antibody responses previously (Dolatshahi et al., 2022a).

### Maternal HIV infection is associated with selective Fc-receptor-dependent antibody transfer changes

**Figure 4:**
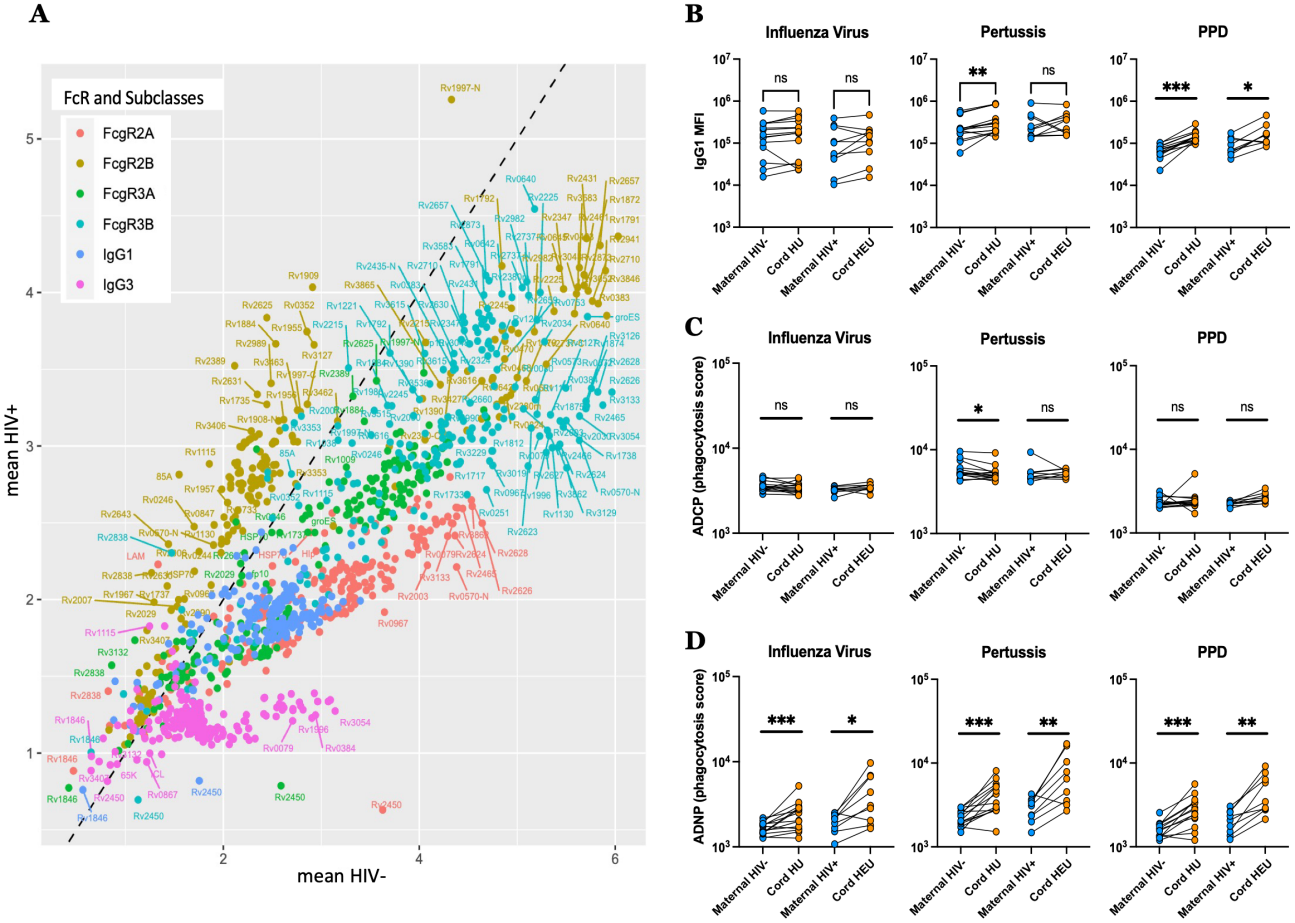
Distinct transfer of *Mtb*-specific antibodies to the umbilical cord blood in HIV-infected and -uninfected individuals. **(A)** Scatter plots showing the mean of the transfer ratio for antibody levels and their capacity to bind to FcgR, with the HIV positive pregnancies plotted on the y-axis, and the HIV negative pregnancies on the x-axis. The dashed line corresponds to similar transfer between HIV negative and positive pregnancies. **(B–D)** The dot plots show IgG1 levels **(B)**, ADCP **(C)** and ADNP **(D)** against Influenza virus, pertussis and PPD in both HIV-infected/exposed and HIV-uninfected/unexposed dyads. Significance was calculated by using paired samples Wilcoxon test, *p < 0.05, **p < 0.01, ***p < 0.001.

To gain further insight into the selective changes that may lead to altered antibody transfer in the setting of maternal HIV infection, transfer ratios for each antibody measurement were compared across the maternal groups (Figure 4A). IgG1 was transferred preferentially to IgG3 in both HIV-infected (*y*-axis) and HIV-uninfected (*x*-axis) pregnant people. However, both were transferred more efficiently in HIV-uninfected pregnant people, with several *Mtb*-specific IgG3 antibodies showing disproportionately low transfer in HIV-infected people. Moreover, particular Fc-receptor-binding antibodies showed preferential transfer in both HIV-infected and -uninfected groups. Specifically, for most antibody parameters, enhanced transfer was noted in HIV-uninfected people, with a small group of FcγR2B features and all FcγR3 binding antibody analytes demonstrating the highest transfer potential. Conversely, only a subset of *Mtb*-specific FcγR2B-binding antibodies were enriched in the HIV-infected group. These data point to a strict FcγR mediated sieving through the placenta, whereby FcγR2B/FcγR3 may selectively draw subsets of antibodies out of the plasma in HIV-uninfected pregnant people to deliver immunity more effectively to neonates. In contrast, during maternal HIV infection, with co-occurring hypergammaglobulinemia and inflammation, the placenta may adapt and reduce antibody transfer, reaching umbilical cord levels similar to those of HIV-uninfected/unexposed dyads, which may prevent hypergammaglobulinemia in the newborn. This appears to be achieved via reduced general IgG1/IgG3 and Fc-receptor mediated transfer, with the exception of a subset of *Mtb*-specificities that are transferred preferentially in HIV-infected people via FcγR2B, which is expressed on placental Hofbauer cells (Martinez et al., 2018). Given the inhibitory nature of FcγR2B binding, this unique antibody transfer may lead to transfer of dampened functional immunity to HEU children, potentially rendering them more vulnerable to infections like TB.

Moreover, altered transplacental transfer was further observed across both non-*Mtb* and *Mtb* antigen specificities in HIV-infected/exposed dyads (Figures 4B–D). For example, while Influenza hemagglutinin specific IgG1 levels were transferred at equal rates across HIV-positive and negative women/cord dyads (Figure 4B), with antibody dependent cellular monocyte-phagocytic (ADCP) inducing antibodies that were also transferred equally (Figure 4C), antibody dependent neutrophil phagocytic (ADNP) antibodies were preferentially transferred in both groups, with a higher fold increase in umbilical cord observed in the HIV-negative/unexposed group, confirming selective transfer of particular antibody effector functions across the placenta. Collectively, these data point to placental antibody sieving, based on significant changes in placental transfer of FcγR binding in the setting of maternal HIV infection, that may adapt in the setting of hypergammaglobulinemia to transfer TB and non-TB-specific antibodies to the newborn.

### Maternal HIV viral load and CD4 count are major drivers of *Mtb*-specific antibody transfer across the placenta

**Figure 5:**
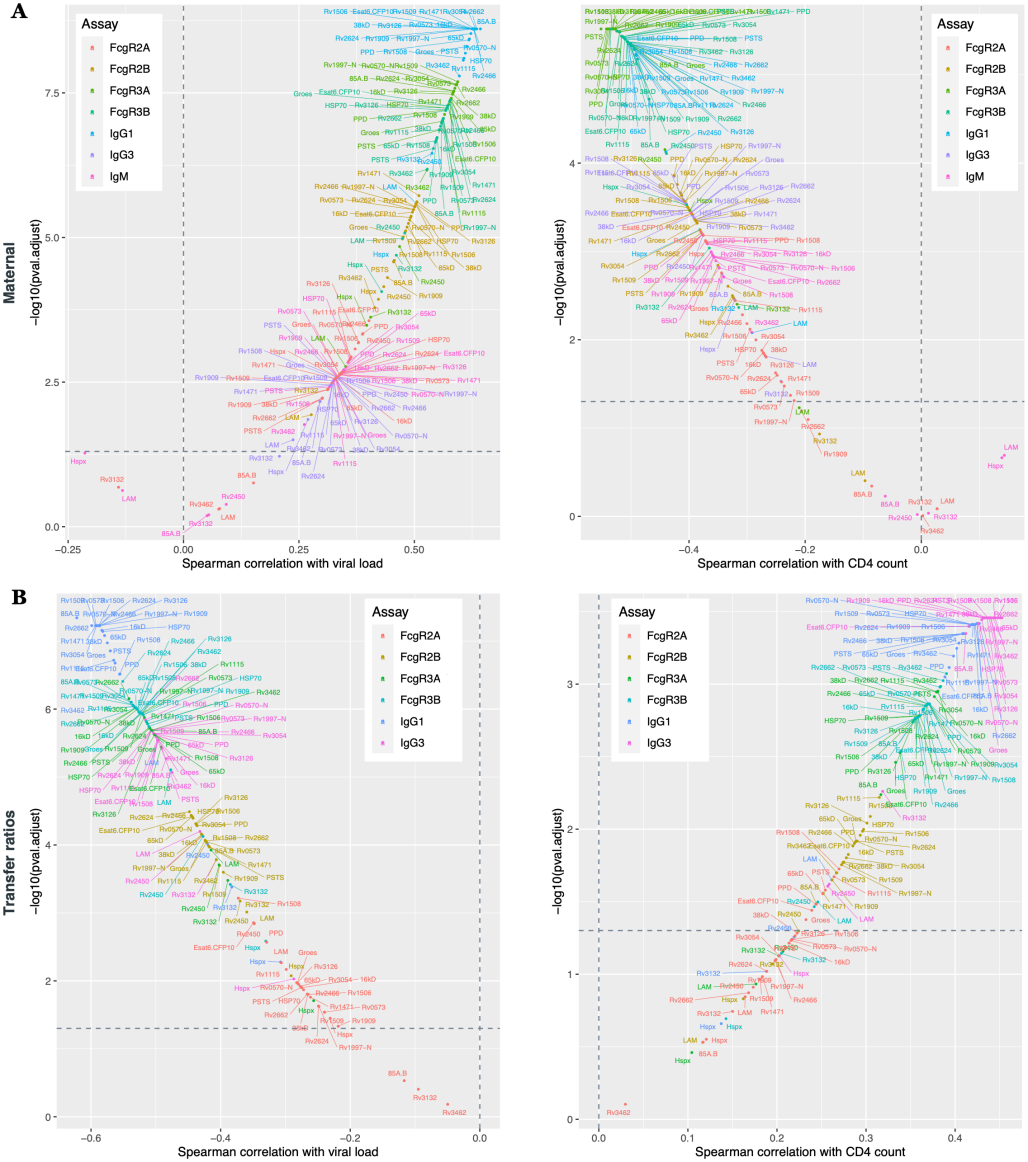
Maternal HIV viral load and CD4 count cells significantly impacts *Mtb*-specific antibody transfer through the placenta. Scatter plots show the Spearman correlations between *Mtb*-specific antibodies and maternal viral load and CD4 count. Antibody response from the maternal plasma **(A)** and transfer ratios **(B)** are illustrated. *Y*-axis represents –log10 (adjusted *p* value), *x*-axis represent the Spearman correlations. Values above the horizontal dashed lines are associated with a significant correlation (*p* < 0.05).

Given the differences in antibody profiles in maternal plasma (Figures 1, 2), umbilical cord blood (Figures 1, 2) and in placental antibody transfer signatures (Figures 3, 4) between HIV-infected and -uninfected pregnant people, we next aimed to determine whether particular clinical markers of immune health in HIV-infected people were associated with alterations in *Mtb*-specific antibody transfer, as we previously observed for herpesvirus and vaccine-specific antigens (Dolatshahi et al., 2022b). Maternal HIV viral load was significantly associated with higher levels of IgG1, able to bind to several Fc-receptors (FcγR3A > FcγR3B > FcγR2B > FcγR2A) (Figure 5A). Interestingly, CD4 count was associated with the opposite effect, such that lower CD4 count was associated with a highly significant reduction in *Mtb*-specific antibody profiles, marked by reduced FcγR3A-and FcγR3B-binding IgG1 antibodies and reduced overall IgG3 and IgM responses (Figure 5B). Moreover, placental antibody transfer was also significantly affected by HIV viral load and CD4 count, but in a more uniform hierarchical manner, with significantly lower IgG1 > FcγR3A/FcγR3B-binding > IgG3 and then FcγR2B and FcγR2A binding antibody transfer. Overall, these data reveal specific defects in *Mtb*-specific antibody transfer in the setting of maternal HIV infection, related primarily to reduced FcγR3-IgG1 transfer across the placenta. Thus, these data point to HIV viremia and lower CD4 count as major drivers of reduced transplacental antibody transfer that may render newborns in TB-endemic regions highly vulnerable to *Mtb* infection.

## Discussion

Neonates are more vulnerable than adults to infections by a range of pathogens, including *Mtb* infection that causes significant morbidity and mortality (Newton et al., 2008; Basu Roy et al., 2019). Specifically, while TB progression occurs in ~5–10% of adults, *Mtb* infection in infants much more frequently results in disseminated forms of TB including miliary TB and TB meningitis, both associated with poor prognosis (Basu Roy et al., 2019) and occurring more frequently in infants born to HIV-infected people (Madhi et al., 2011; Ruck et al., 2016). In newborns, the immature nature of the innate and adaptive immune response, coupled to immature respiratory barrier function and simultaneous microbe commensalization, have all been implicated in enhanced susceptibility to TB progression (Newton et al., 2008; Liu et al., 2017; Olin et al., 2018), and are all modulated by maternal HIV infection (Adhikari et al., 2011; Xu et al., 2022). However, beyond barrier and cellular immune defenses, emerging data point to a critical role for antibodies in the control of *Mtb*. Specifically, *Mtb*-specific antibodies from latently infected individuals restrict *Mtb* growth in macrophages (Lu et al., 2016), and passively transferred antibodies attenuate TB disease and protect against a lethal challenge in mice (Achkar et al., 2015). Furthermore, *Mtb*-specific antibodies are associated with enhanced protection against *Mtb* infection following intravenous vaccination with Bacille Calmette–Guérin (BCG) (Irvine et al., 2021), and *Mtb*-specific antibodies exhibit different specificities and functions in the setting of HIV infection (Bell and Noursadeghi, 2018; van Woudenbergh et al., 2020; Nziza et al., 2022). However, whether *Mtb*-specific transplacental antibody transfer differs between HIV-infected and -uninfected pregnant people and to what degree this transfer explains vulnerability *to Mtb* in early life remains unknown. Here, we observed significant differences in *Mtb*-specific humoral immune profiles in HIV-infected and -uninfected pregnant people, likely due, in part, to HIV-associated inflammation and hypergammaglobulinemia (De Milito et al., 2004). However, overall antibody profiles in the umbilical cord were more similar, pointing to placental normalization of specific antibodies transferred to umbilical cord blood. Granular analysis of antibody transfer profiles pointed to critical, and proportional, antibody sieving across the placenta in both maternal HIV subgroups, with reduced transfer of individual IgG subclass and Fcγ-receptor binding populations. However, a subset of FcγR2B-binding antibodies did exhibit preferential transfer in HIV-infected/exposed dyads. These data point to an unexpected mechanism of passive antibody transfer regulation, that may regulate antibody transfer in the setting of hypergammaglobulinemia, but potentially lead to some abnormal transfer of anti-inflammatory FcγR2B-binding responses in the setting of HIV.

While emerging data point to a role for additional Fc-receptors, beyond the neonatal Fc-receptor (FcRn), in regulation of placental antibody transfer (Martinez et al., 2019; Dolatshahi et al., 2022a), the precise mechanism of placental antibody transfer remains incompletely defined. While strong relationships exist between the level of antibodies in maternal plasma and newborn plasma (Kohler and Farr, 1966; Palmeira et al., 2012), selective transfer of particular antibody subpopulations (Clements et al., 2020) may be transferred by activating Fc-receptors that may capture and co-translocate antibodies with the neonatal Fc-receptor (Jennewein et al., 2019). Placental integrity, maternal health, and the type of antigens have all been proposed as determinants that dictate antibody transfer dynamics (Palmeira et al., 2012). Yet, understanding the specific rules that govern antibody transfer in the setting of health and disease could provide critical insights for the design of more effective therapeutics and vaccines to protect both the pregnant person and newborn. Specifically, whether inflammatory disease-induced changes in Fc-glycosylation and/or placental Fc-receptor expression alter antibody transfer is unknown. However, here we observed a significant shift in the quality (including isotype and antigen specificities, as well as Fc-effector functions) of antibody transfer in the setting of HIV-infection toward reduced inflammatory antibody transfer, which has also been described with other pathogens, including herpesvirus, tetanus, and poliovirus (Dolatshahi et al., 2022a). Given that infections drive the production of inflammatory antibodies, poised to rapidly control and clear pathogens, it is plausible that the placenta may respond to the same systemic inflammatory cascades and alter Fc-receptor expression to prevent the transfer of antibodies that may cause pathologies in infants. Similarly, previous studies observed differential Fc-receptor expression in the placenta of SARS-CoV-2 infected compared to uninfected people (Atyeo et al., 2021), arguing that the placenta is a dynamically adapting organ that induces the most appropriate antibody transfer for the newborn. Along these lines, placental adaptation has also been observed throughout gestation, with preferential transfer by distinct Fc-receptors earlier in pregnancy (Dolatshahi et al., 2022a), populating infants with highly functional antibodies even when born preterm. Here, we observed a correlation between antibody transfer profiles and maternal CD4 count and HIV viral load in HIV-infected pregnant people, further supporting the impact of systemic-immunologic inflammatory changes on placental activity. However, remarkably, this placental adaptation resulted in similar antibody profiles in both maternal HIV groups, suggesting that the placental adaptations aim to normalize and deliver the same quantity and quality of antibodies to infants irrespective of the inflammatory state of the mother. However, whether enhanced transfer via the sole inhibitory Fc-receptor, FcγR2B, has additional functional consequences in the infant, remains unclear, but could point to down-stream vulnerabilities in antibody functionality that may account for the higher risk of TB disease in HEU infants (Madhi et al., 2011; Ruck et al., 2016).

HEU infants have a higher risk of developing several infections (Ruck et al., 2016), including TB (Madhi et al., 2011). This project provides clues related to the differential transfer of *Mtb*-specific antibodies across the placenta, and points to the remarkable adaptive activity of this unique organ, aimed at regulating and transferring antibodies of the highest value to infants to provide protection in the first days of life. While sufficient umbilical cord plasma was not available to profile the functional glycan-specific antibody quality, future studies focused on further defining the effector profiles of the transferred antibodies as well as how they interact with innate immune cells from the HEU and HU infants may provide further insights into the immune vulnerabilities rendering children vulnerable to *Mtb*. Moreover, integrating insights on the unique nature of placental transfer biology in vaccine development may lead to the generation of new vaccines able to provide enhanced protection against different infectious diseases, not only TB, in infants at a global level. Additional studies should also assess the impact of HIV on antibody transfer in milk as well as the impact of maternal HIV status on the evolution of immunity beyond infancy in order to understand the long-term effect of maternal HIV infection on their developing immune system.

## Data availability statement

The raw data supporting the conclusions of this article will be made available by the authors, without undue reservation.

## Ethics statement

The studies involving human participants were reviewed and approved by Mbarara University of Science and Technology (11/03-17) Partners Healthcare (2017P001319). The patients/participants provided their written informed consent to participate in this study.

## Author contributions

GA and NN designed the study. NN and KF performed the experiments. NN, WJ, and MM performed the computational analysis. LB collected the samples and managed clinical data. NN, LB, and GA drafted the manuscript, with contributions from RM, SF, TO, and BB. All authors contributed to the article and approved the submitted version.

## Funding

This work was supported by gates Foundation (OPP1159416, OPP1151840, and OPP1156795) and the National Institutes of Health (1R561AI155149, the IMPACTB Consortium, and 3R37AI080289-11S1 to GA). It was also supported by the Harvard University Center for AIDS Research National Institutes of Health/National Institute of Allergy and Infectious Diseases (grant number P30AI060354 to LB) and supported by a KL2/Catalyst Medical Research Investigator Training award from Harvard Catalyst | The Harvard Clinical and Translational Science Center (grant number KL2TR002542 to LB), and the Charles H. Hood Foundation (to LB), a career development award from the National Institute of Allergy and Infectious Diseases (grant number K23AI138856 to LB), and the American Society of Tropical Medicine and Hygiene Burroughs Wellcome Postdoctoral Fellowship in Tropical Infectious Diseases (to LB). The sponsors had no role in study design, data collection, analysis, or interpretation, writing the report, or decision to submit the article for publication. The content is solely the responsibility of the authors and does not necessarily represent the official views of Harvard Catalyst, Harvard University and its affiliated academic healthcare centers, the National Institutes of Health, or other funders.

## Conflict of interest

GA is a V.P. at Moderna, a founder and equity holder of SeromYx Systems, and an employee and equity holder of Leyden Labs. GA’s interests were reviewed and are managed by MH and Partners HealthCare in accordance with their conflict-of-interest policies.

The remaining authors declare that the research was conducted in the absence of any commercial or financial relationships that could be construed as a potential conflict of interest.

## Publisher’s note

All claims expressed in this article are solely those of the authors and do not necessarily represent those of their affiliated organizations, or those of the publisher, the editors and the reviewers. Any product that may be evaluated in this article, or claim that may be made by its manufacturer, is not guaranteed or endorsed by the publisher.
