# Supplementary material for: Maternal HIV infection drives altered placental Mtb-specific antibody transfer
Source: Front Microbiol. 2023 May 9;14:1171990. doi: 10.3389/fmicb.2023.1171990 (PMC10203169; doi:10.3389/fmicb.2023.1171990)
Supplement: Supplementary file 1 [file Data_Sheet_1.PDF]

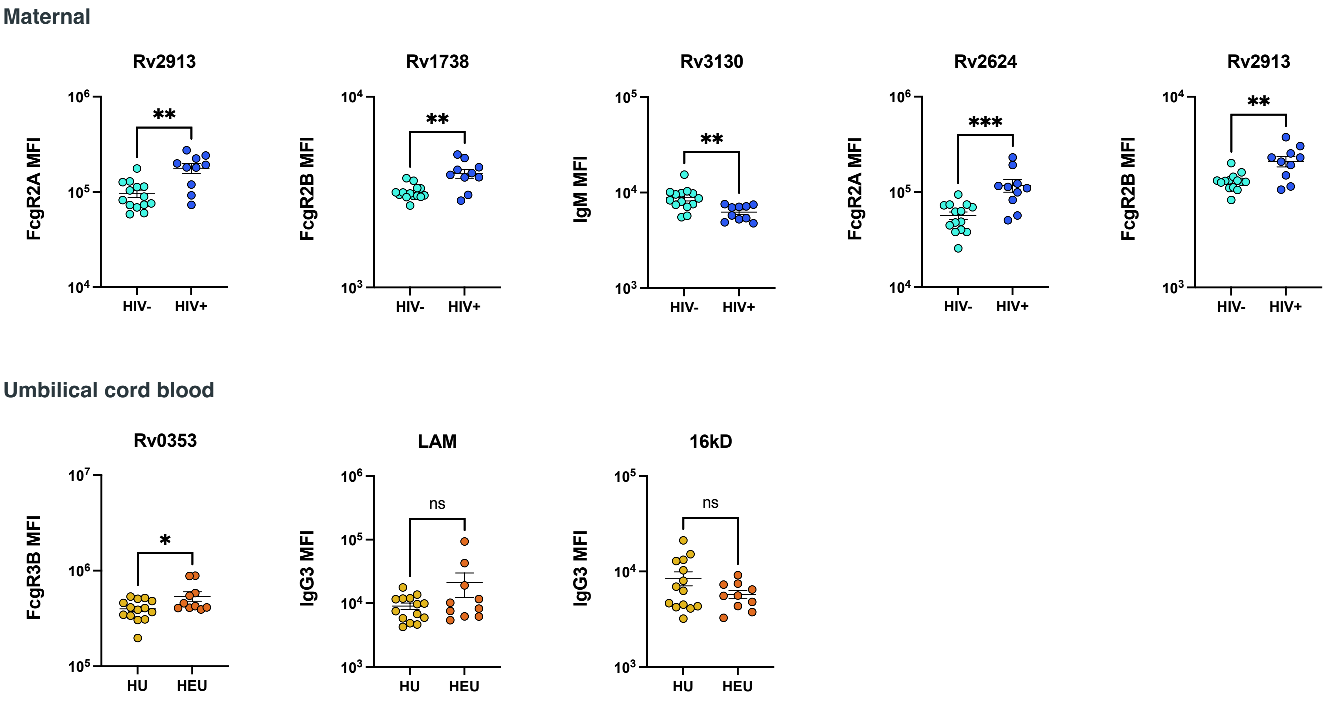

Supplemental FIGURE 1. Differences between antibody profile in HIV-infected/exposed and HIV-uninfected/unexposed dyads.
